# Injectable Hydrogel With Glycyrrhizic Acid and Asiaticoside‐Loaded Liposomes for Wound Healing

**DOI:** 10.1111/jocd.16606

**Published:** 2024-10-02

**Authors:** Yunqi Zhang, Yu Xiong, Xiaochun Wu, Maofang Huang, Zhengjie Li, Tie Zhao, Peng Peng

**Affiliations:** ^1^ Department of Pharmacy Guangzhou Dermatology Hospital Guangzhou China; ^2^ Orthopedics Department Guangdong Provincial Second Hospital of Traditional Chinese Medicine Guangzhou China; ^3^ Guangdong Provincial Engineering Technology Research Institute of Traditional Chinese Medicine Guangzhou China; ^4^ Guangdong Provincial Key Laboratory of Research and Development in Traditional Chinese Medicine Guangzhou China

**Keywords:** asiaticoside, GelMA/Lip@GA@AS hydrogel, glycyrrhizic acid, wound healing

## Abstract

**Background:**

Open skin wounds increase the risk of infections and can compromise health. Therefore, applying medications to promote healing at the injury site is crucial. In practice, direct drug delivery is often difficult to maintain for a long time due to rapid absorption or wiping off, which reduces the efficiency of wound healing. Consequently, the development of bioactive materials with both antibacterial and wound‐healing properties is highly desirable.

**Methods:**

This study synthesized liposomes loaded with glycyrrhizic acid (GA) and asiaticoside (AS) by film dispersion‐ultrasonication method, which were then incorporated into a GelMA solution and cross‐linked by ultraviolet light to form a bioactive composite hydrogel for wound dressings.

**Results:**

This hydrogel is conducive to the transport of nutrients and gas exchange. Compared with GelMA hydrogel (swelling rate 69.8% ± 5.7%), the swelling rate of GelMA/Lip@GA@AS is lower, at 52.1% ± 1.0%. GelMA/Lip@GA@AS also has better compression and rheological properties, and the in vitro biodegradability is not significantly different from that of the collagenase‐treated group. In addition, the hydrogel polymer has a stable drug release rate, good biocompatibility, and an angiogenic promoting effect. In vitro experiments prove that, at concentrations of 0.5, 1, 2, and 3 mg/mL, GelMA/Lip@GA@AS can inhibit the growth of *Staphylococcus aureus*.

**Conclusion:**

We synthesized GelMA/Lip@GA@AS hydrogel and found it possesses advantageous mechanical properties, rheology, and biodegradability. Experimental results in vitro showed that the bioactive hydrogel could efficiently release drugs, exhibit biocompatibility, and enhance angiogenesis and antimicrobial effects. These results suggest the promising application of GelMA/Lip@GA@AS hydrogel in wound‐dressing materials.

## 1 Introduction

Skin injuries are inevitable in daily life. Once the wound area is relatively large or deep, medical intervention is necessary to prevent infection and help healing. Glycyrrhizic acid (GA) is a triterpenoid compound extracted from the roots and rhizomes of licorice. It is a major bioactive component with antiviral, anti‐inflammatory, and hepatoprotective properties [1, 2, 3]. It has also been reported to be used in psoriasis [4], subcutaneous tissue inflammation [5], and improved drug delivery [6]. GA shares an anti‐inflammatory mechanism similar to hydrocortisone, as both target critical enzymes like cyclooxygenase and phospholipase A2 involved in inflammation. This action leads to a direct reduction in prostaglandin synthesis, thereby impeding inflammation and concurrently inhibiting platelet aggregation [7]. Additionally, GA exhibits the capability to hinder reactive oxygen species (ROS) production by neutrophils at sites of inflammation [8]. Asiaticoside (AS) is primarily isolated from safflower and is a triterpenoid saponin compound, exhibiting a wide range of biological activities including antioxidant, anti‐inflammatory, anti‐apoptotic, neuroprotective, moisturizing, and wound healing effects [9, 10]. Currently, AS is widely used in cosmetology and is advertised to have anti‐photoaging capabilities [11] and avoid scarring [12]. Some studies show that AS can also promote angiogenesis [13, 14]. Others have found that AS could induce human collagen I synthesis through TGFβ receptor I kinase (TβRI kinase) non‐dependent Smad signaling [15]. Similar to GA, AS can also exert anti‐inflammatory effects by inhibiting the activity of the pro‐inflammatory enzyme phospholipase A2 [16]. By combining both their anti‐inflammatory properties and the wound healing effects of AS, it enhances wound healing, reduces infection and inflammation, and improves skin regeneration. Therefore, it is valuable to investigate delivery materials capable of stably and effectively releasing two medicinal components at the targeted site.

Multiple studies have defined that encapsulation of drugs in liposomes can significantly slow down the clearance rate of drugs and alter the relative distribution of drugs in tissues [17, 18, 19]. GA‐loaded or AS‐loaded liposomes have also been utilized for inflammatory wound healing [20, 21]. However, there are some issues such as wound infection and scarring that need to be taken seriously. Hydrogels are hydrophilic three‐dimensional network materials with biophysical properties. Currently, antimicrobial hydrogels have been developed to replace existing antimicrobial agents, offering advantages such as low cost, ease of synthesis, good stability, localized release, avoidance of repeated dosing, good biocompatibility, and promotion of tissue repair [22]. Methacrylated gelatin (GelMA) has become a representative hydrogel formulation and has garnered extensive utilization in diverse biomedical applications [23].

Due to the fragility of capillaries and the response of the immune system, bleeding and other body fluid exudation often occur when the skin is injured, so water absorption is an important indicator of hydrogels. The absorption of exudate by hydrogels helps keep the wound clean and is also beneficial for drug release and wound healing [24]. However, excessive absorption of water can cause structural damage, breakage, or shedding [25]. Liposome‐loaded hydrogel has better stability [26] and anti‐swelling ability based on multiple cross‐linking [27] and it can avoid substantial initial burst drug release and extend the duration of use [28, 29].

In this study, we synthesized a GelMA/Lip@GA@AS hydrogel and characterized its structural properties. It is worth noting that the liposome‐loaded hydrogel improves the mechanical properties of GelMA and has better swelling behavior. The incorporation of GA and AS gives the hydrogel the ability to promote angiogenesis and antibacterial properties in vitro. In addition, GelMA/Lip@GA@AS has good biocompatibility and has potential value in medical applications.

## 2 Experimental Section

### 2.1 Materials and Reagents

Teleostean gelatin (Gel) was obtained from Sigma‐Aldrich (St. Louis, MO, USA). Lecithin from soybean, cholesterol, GA, AS, and methacrylic anhydride (MA) were purchased from Shanghai Macklin Technology Corp (Macklin, Shanghai, China).

### 2.2 Synthesis of Liposomes

Liposomes were prepared following a film dispersion‐ultrasonic method. Soybean lecithin (30 mg), cholesterol (5 mg), and GA (17 mg) were dissolved in 40 mL ethyl acetate solution and evaporated into a uniform film. After the organic solvent was removed by vacuum evaporation at 40°C, 17 mL PBS containing AS (1 mg/mL) was added. After ultrasonic 5 min, the organic solvent was rotated for 2 h at room temperature. Then, the liposome loaded with GA and AS (Lip@GA@AS) was obtained by ultrasonic 2 min (600 W, working 3 s, pause 1 s) under the ice bath. The whole preparation process of blank Liposomes (Lip) was the same as that of Lip@GA@AS except that GA and AS were not added. After high‐speed centrifugation, the supernatant of Lip@GA@AS was diluted with methanol gradient. The concentration in the supernatant was quantified according to the pre‐established standard curve. The encapsulation efficiency (EE) and drug loading (DL) in Lip@GA@AS were calculated according to the formula:

$$\mathrm{EE}\;\left(\%\right)=\left(W_{1}-W_{2}/W_{1}\right)\times 100\%$$

*W*_1_ and *W*_2_ were the weights of added content and unloaded content, respectively.

$$\mathrm{DL}\;\left(\%\right)=\left(W_{E}/W_{P}\right)\times 100\%$$

*W*_E_ and *W*_P_ were the weights of encapsulated drug and Lip@GA@AS liposomes.

### 2.3 Synthesis of Methacrylic Acid Gelatin (GelMA)

10‐g Gel was dissolved in PBS at 50°C of 100 mL; then, 6 mL MA was slowly added and reacted at 50°C for 12 h. Then, the mixed solution was dialyzed (MWCO 12 000–14 000 Da) in distilled water for 3 days, with water changes every 2 h, to remove the unreacted monomers. The dialyzed solution was lyophilized to obtain GelMA.

### 2.4 Characterization of Liposomes

The appearance and distribution of Lip and Lip@GA@AS were observed by transmission electron microscope (TEM, H7700; Hitachi, Japan). Lip and Lip@GA@AS were dripped on the copper net of the Formwan membrane (the membrane faces up). After 5 min, we used a filter paper to absorb the excess liquid from the edge of the copper net. Then, 1% phosphotungstic acid dye solution was dripped into the copper mesh, and after 1 min, the remaining liquid was also absorbed. The copper net was dried and imaged by TEM. The particle sizes of Lip and Lip@GA@AS were measured by Zeta‐sizer (ZSU570; Malvern NanoZS, UK).

### 2.5 Hydrogel Preparation and Characterization

0.5‐g GelMA was dissolved in 10 mL PBS (containing 0.1% LAP) to prepare 10% GelMA solution, then a certain amount of Lip@GA@AS was added, and finally GelMA/Lip@GA@AS hydrogel was cross‐linked by UV irradiation. The chemical structures of Gel and GelMA were determined by nuclear magnetic resonance (^1^H NMR) spectrometer (AVANCE III 500 M; Bruker, Germany) utilizing a mixture of D_2_O as the solvent. The Fourier transform infrared spectra (FTIR) of Gel and GelMA were recorded by FTIR (Thermoscience NicoletiS20, Waltham, MA, USA). The wave number range was set from 550 to 4000 cm^−1^, the scanning speed was 0.2 cm/s, and the spectral resolution was 4 cm^−1^. The freeze‐dried hydrogel was observed by scanning electron microscope (SEM, SU8010; Hitachi, Japan) to analyze its internal morphology. The average pore size of hydrogel was calculated from SEM images by Nanomeasure software.

### 2.6 Swelling Characterization

Test hydrogels (400 μL) were immersed in a centrifuge tube containing 20 mL PBS solution, then cultured in a constant temperature culture oscillator at 37°C. At different points in time (1, 3, 6, 12, 18, and 24 h), the excess water on the surface of the swollen hydrogels was blotted dry with filter paper, and then, the hydrogels were reweighed. The swelling degree of the composite hydrogel is calculated according to the following formula:

$$\text{Swelling ratio}\;\left(\%\right)=\left(W_{t}-W_{0}\right)/W_{0}\times 100\%$$

*W*_0_ and *W*_t_ are the weight of the tested hydrogel before and after swelling, respectively.

### 2.7 Rheological Characterization

The storage modulus (G′) and loss modulus (G″) of hydrogel were measured by rheometer. The composite hydrogel of 200 μL was placed between the parallel plates, the interval was 2 mm. Time scan (5 rad/s, strain 1%, 300 s) and dynamic strain frequency scan (0.1–100 Hz) were recorded, respectively.

### 2.8 Compression Characterization

The mechanical property of hydrogel samples was tested by universal testing machine (DPS‐5D; Chengyu Testing Equipment, China). The sample was first treated with a preload force of 0.06 N load cell, and then under a cyclic compression frequency of 1 mm/min until fracture. The compression modulus was calculated from the slope of the data of the obtained stress–strain curve between 40% and 60% strain.

### 2.9 In Vitro Degradation Test

The degradation of hydrogels in phosphate buffer (PBS) and collagenase I solution (100 U/mL) was evaluated, which was characterized by monitoring the weight change of hydrogel during soaking. First, the hydrogel was freeze‐dried and weighed (*W*_0_). The hydrogel samples were placed in a six‐well plate containing PBS and collagenase I solution, sealed and placed in a constant temperature shaker at 37°C and 70 rpm. The hydrogel samples were taken out and washed with ultrapure water at each time point of measurement. After freeze‐drying, the hydrogel samples were weighed as (*W*_t_). The weight remaining ratio of the hydrogel is calculated according to the following formula:

$$\text{Weight remaining ratio}\;\left(\%\right)=W_{t}/W_{0}\times 100\%$$

### 2.10 In Vitro GA and AS Release

The in vitro drug release of GeLMA/Lip@GA@AS hydrogel was investigated by dynamic dialysis. GA is an insoluble drug, which can form micelles with it by adding a certain amount of surfactant to increase the solubility of the drug, so 0.9% NaCl solution containing 1% SDS was chosen as the release medium. Unlike GA, AS is a water‐soluble drug, 0.9% NaCL solution was used as the release medium. Take GelMA/Lip@GA or GelMA/Lip@AS in a pretreated dialysis bag (Mw = 10 kDa), add 1 mL of release medium, put it into 50 mL of release medium that has been thermostated to 37°C, and then shaking at 37°C with a constant speed of 100 rpm, respectively. One milliliter of release medium was collected at the specified time points; at the same time, the same amount of release medium was added, and the mass concentration of GA in the sample was determined by UV spectrophotometry. The mass concentration of AS was also taken and measured in the same way as above. In addition, the release procedure of AS was the same as the above experimental procedure, except that the concentration of the release medium was measured by HPLC.

The LIP@AS liposomes and LIP@GA liposomes were dispersed in 2 mL deionized water and transferred to a dialysis bag (MW: 12–14 kDa). Next, the dialysis bag containing samples was incubated in 8 mL of PBS solution in a constant temperature shaker (37°C, 70 rpm). At different time points, the PBS supernatant was collected and replaced with the same volume of PBS solution. The concentration of AS in the collected supernatant was detected by HPLC based on pre‐established standard curve peak area values, and the cumulative release rate was calculated according to the formula:

$$\begin{array}{c} \text{Cumulative release rate}\;\left(\%\right)=\text{Drug release amount}/\\ \;\text{Total drug content}\times 100\% \end{array}$$

### 2.11 Biocompatibility Assessment

To evaluate the biocompatibility of the prepared hydrogels, the cytotoxicity toward 3 T3 mouse fibroblasts was assessed using the CCK‐8 assay. The extraction method involved incorporating the hydrogel at a standard ratio (GB/T 16886.5‐2017) of 0.2 g/mL into DMEM complete culture medium, incubating at 37°C for 24 h to obtain the hydrogel extract. Then, 100 μL of a 3 T3 fibroblast suspension (5 × 10^4^ cells/mL) was seeded in 96‐well plates and incubated in a CO_2_ incubator at 37°C for 1, 2, and 3 days. Following that, CCK‐8 reagent was added to each well, and the incubation continued under the same conditions for 4 h. The control group did not expose to the extract; instead, they were cultured in DMEM (high glucose) containing 10% fetal bovine serum. The absorbance at 450 nm was measured using a microplate reader for both the control (OD_0_) and experimental (OD_1_) groups. The cell viability was calculated using the following formula:

$$\text{Cell survival rate}\;\left(\%\right)={\mathrm{OD}}_{1}/{\mathrm{OD}}_{0}\times 100\%$$

### 2.12 In Vitro Angiogenesis

In vitro tubulogenesis assay was performed using human umbilical vein endothelial cells (HUVECs). The matrix gel was thawed overnight on ice and allowed to solidify in a 48‐well plate at 37°C. HUVECs at a density of 1 × 10^5^ cells/mL were evenly seeded onto the matrix gel, followed by a 4‐h incubation with the hydrogel extract. Under an inverted fluorescence microscope, tubule formation within each well was observed visually.

## 3 Results

### 3.1 Structural Characterization of Polymer Materials

**FIGURE 1:**
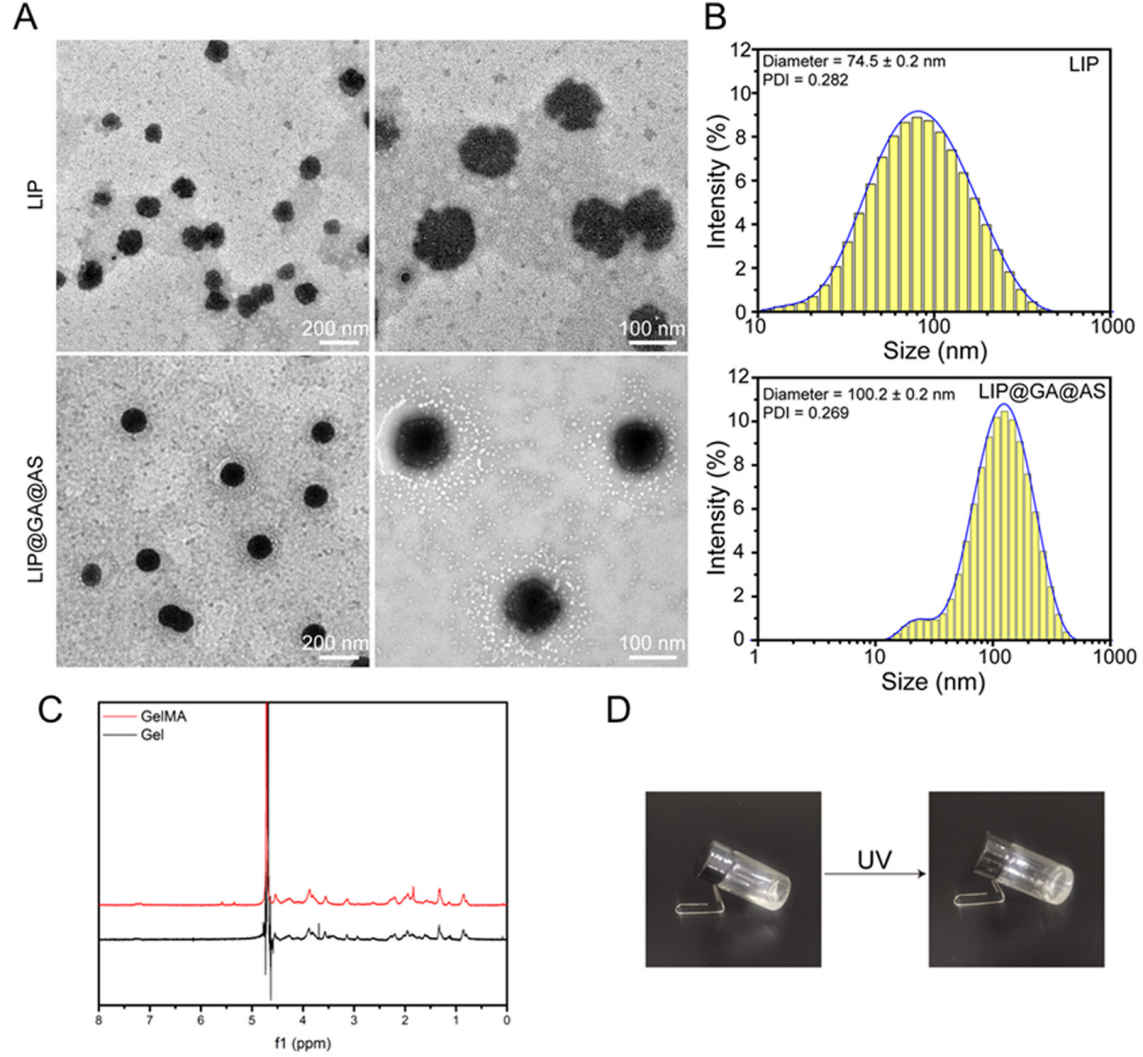
(A) TEM images of Lip and Lip@GA@AS; (B) Particle size distribution of Lip and Lip@GA@AS; (C) NMR hydrogen spectra of Gel and GelMA; (D) Hydrogels formed by the action of UV light and LAP.

The morphology of liposomes was characterized using transmission electron microscopy (TEM). The liposomes exhibited a regular spherical structure with an average size of approximately 80 nm. As depicted in the figures, under the optimal conditions, the Lip@GA@AS liposomes were prepared via thin‐film dispersion and sonication. The morphology of liposomes was uniform, appearing spherical with an average size of approximately 100 nm (Figure 1A). Concurrently, the particle size of Lip@GA@AS was significantly larger than that of LiP, and the surface was also smoother than LiP, which indicates the successful loading of GA and AS. The average particle sizes of Lip and Lip@GA@AS liposomes were determined using dynamic light scattering (DLS) to be 74.5 ± 0.2 nm (PDI 0.282) and 100.2 ± 0.2 nm (PDI 0.269), respectively (Figure 1B), which were consistent with the results shown in TEM images.

MA was used for graft copolymerization of gelatin, converting it into a double bond‐containing GelMA. The chemical structure of GelMA was analyzed using proton nuclear magnetic resonance (NMR) spectroscopy. The peaks observed at 5.36 and 5.6 ppm correspond to the vibrations of the C=C bonds after acrylic acid modification, confirming the successful modification of gelatin with MA (Figure 1C).

Next, the prepared GelMA was mixed with the photoinitiator (LAP), as GelMA possesses –C=C– bonds that can undergo polymerization upon exposure to ultraviolet light in the presence of LAP. Before UV irradiation, the bottle containing the GelMA and LAP mixture was tilted and the liquid surface flowed to be parallel to the ground. After UV irradiation, GelMA solidified under the reaction of LAP, and the liquid turned into a translucent solid. After tilting the bottle, the hydrogel was fixed at the bottom of the bottle and could not flow (Figure 1D).

### 3.2 SEM and Swelling Performance Analysis of the Hydrogel

**FIGURE 2:**
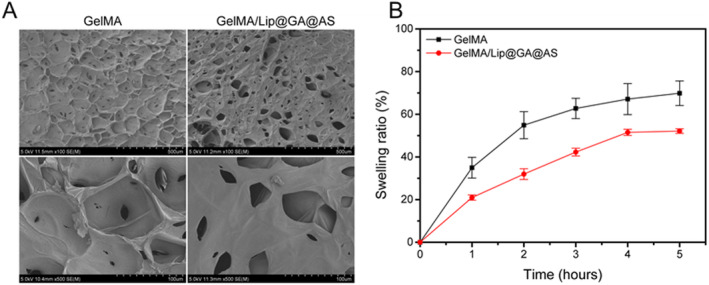
(A) Representative SEM images of different hydrogels; (B) Solubilization properties of different hydrogels.

The scanning electron microscopy (SEM) results depict the microstructure of hydrogels, including pore size, pore arrangement, and interconnectivity between pores. All hydrogels exhibited an irregular three‐dimensional network structure, which could promote the transport of nutrients and gas exchange during the cell culture process. As important structures for drug delivery and penetration, pores affect the diffusion of drugs in hydrogels and the rate of drug release into wounds [30]. We observed that the hydrogel loaded with liposomes exhibited a greater number of pores compared to GelMA, and these pores were also larger in size to give hydrogel a larger surface area (Figure 2A). These pores help the hydrogel adhere firmly to the wound. The larger surface area is conducive to the exchange of drugs and wound exudate and maintains good air permeability.

The hydrogel absorbs wound exudate, but continuous absorption leads to swelling, causing it to detach and lose effectiveness, which is a common issue with conventionally prepared hydrogels, and poses challenges in various biomedical applications such as soft actuation, tissue engineering, bioelectronics, and internal wound closure. This swelling typically results in volume expansion, compromising the mechanical integrity of the hydrogel and potentially exerting unwanted pressure on surrounding tissues when used in vivo [27]. Therefore, balancing the hydrogel's water absorption capacity and structural damage caused by swelling is crucial to enhance the effectiveness and safety of hydrogel applications in biomedical settings. We also analyzed the swelling rates of the different hydrogels in aqueous solutions. The swelling curves indicate that both GelMA and GeLMA/Lip@GA@AS hydrogels reach equilibrium swelling within 5 h, with maximum swelling ratios of 69.8% ± 5.7% and 52.1% ± 1.0%, respectively (Figure 2B). This indicates that GeLMA/Lip@GA@AS has higher mechanical strength while maintaining its water absorption capacity.

### 3.3 Physiochemical Properties and In Vitro Drug Release Performance of the Hydrogel

**FIGURE 3:**
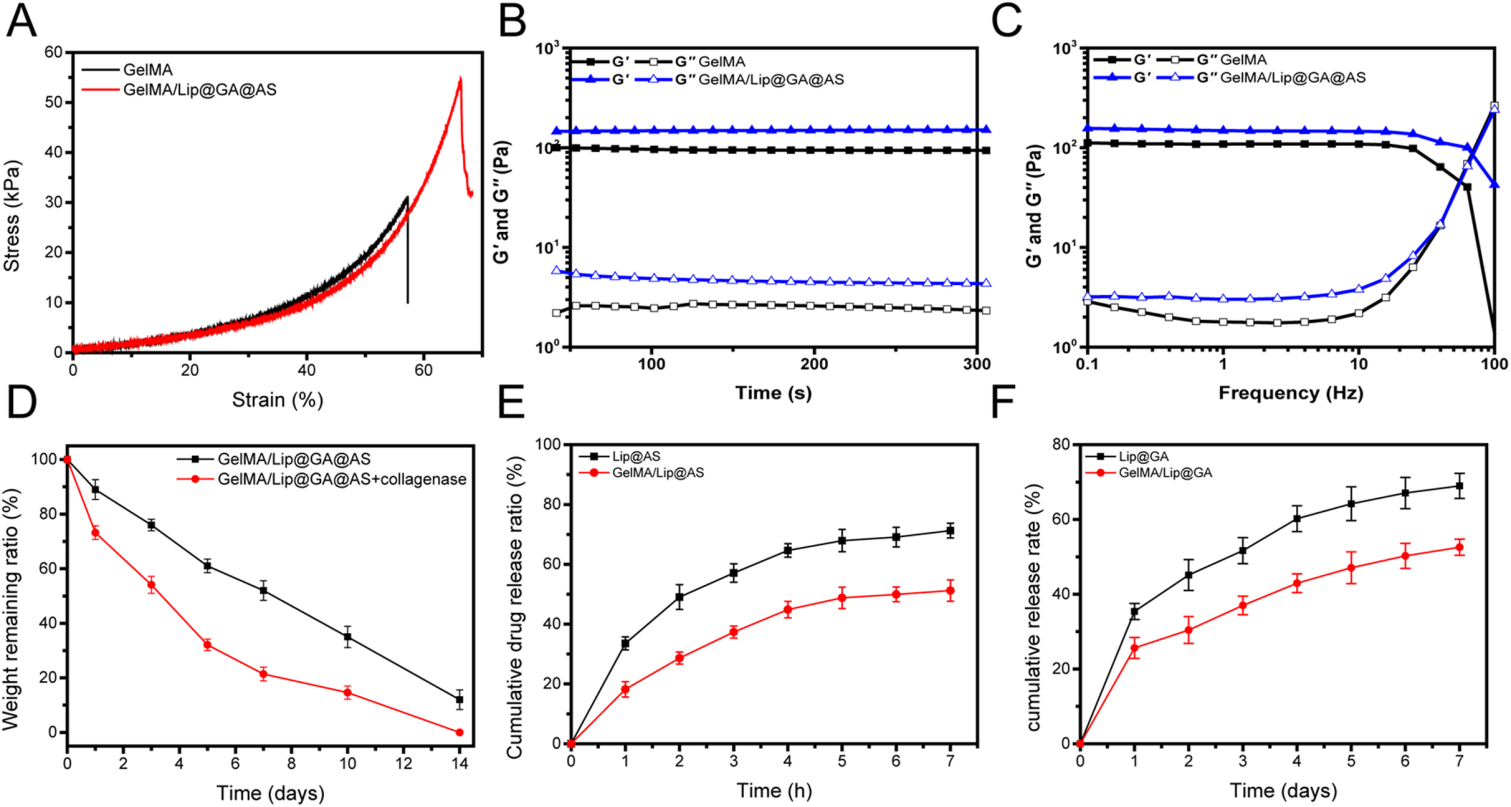
(A) Stress‐strain curve of hydrogel; (B) Plot of G′ and G″ versus time for hydrogel materials; (C) Plot of hydrogel materials G′, G″ versus frequency; (D) In vitro degradation of hydrogel under natural conditions and collagenase; (E, F) Plots of drug release profiles of GA and AS in vitro.

An ideal hydrogel should possess excellent mechanical properties to ensure convenience and integrity during application. The results indicate that GelMA/Lip@GA@AS hydrogel exhibited the highest stiffness. In comparison, GelMA hydrogel was softer, with rupture occurring around 55% compression (Figure 3A). The maximum compressive stress for GelMA and GelMA/Lip@GA@AS hydrogels were 57.2 and 66.3 kPa, respectively. Liposomes have their own structural integrity. When dispersed in a hydrogel matrix, they can act as a microscopic skeleton to enhance the overall stiffness and elasticity of the hydrogel. Besides, the rheological properties of the hydrogel material, as depicted by the graphs showing the changes in storage modulus (G′) and loss modulus (G″) with time, indicate that neither G′ nor G″ exhibits significant increases over time. Moreover, G′ consistently remains greater than G″, suggesting good stability of the hydrogel (Figure 3B). Upon gelation, G′ remains higher than G″ for all frequencies, demonstrating that the hydrogel maintains its gel state stably (Figure 3C).

We hope that this hydrogel polymer will not be easily degraded in vitro, which will ensure continuous and efficient action at the site of action. Therefore, we investigated the in vitro degradability of this hydrogel polymer. We found that freeze‐drying and weighing after immersion in a PBS solution containing 100 U/mL collagenase over a 14‐day timeframe did not result in a significant difference in weight compared with the hydrogel that had undergone freeze‐drying alone, suggesting that this hydrogel polymer was not easily degraded (Figure 3D).

The drug‐carrying properties of hydrogel liposomes are very important. Stabilizing the drug load and releasing it slowly can improve the duration of drug action and therapeutic efficacy. When released individually, GA and AS in Lip@GA@AS exhibited a sudden release pattern. However, the release profiles of GA and AS from GelMA/Lip@GA@AS showed a significantly slower release rate, with the drug concentration remaining constant over time (Figure 3E,F). These findings indicate that GelMA hydrogel plays a role in drug release, preserving the integrity of liposomes and enhancing their stability.

### 3.4 In Vitro Evaluation of Biocompatibility and Angiogenesis

**FIGURE 4:**
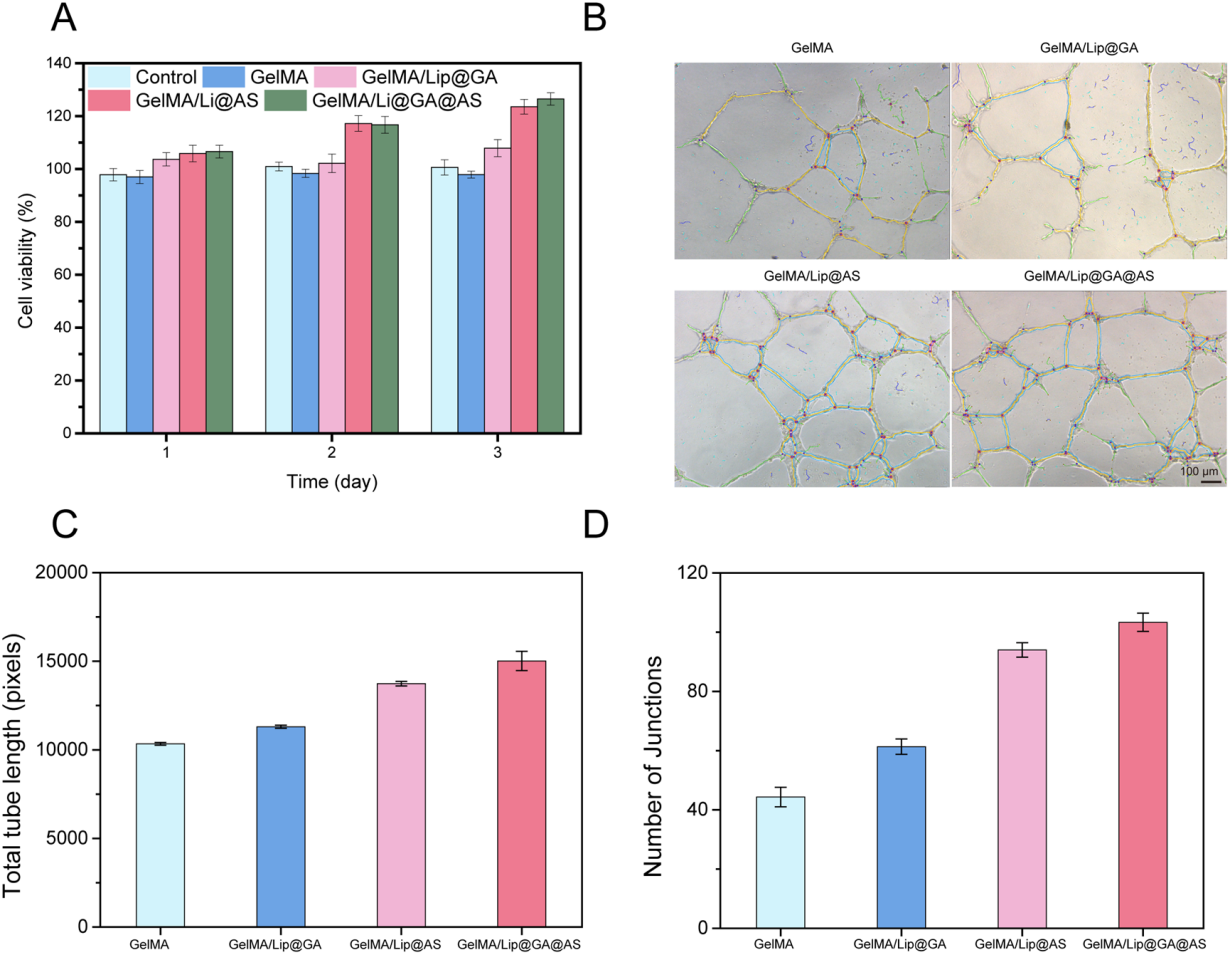
(A) In vitro compatibility evaluation of four hydrogels; (B) Representative images of in vitro angiogenesis of hydrogels; (C) Quantitative data on total tube length of four hydrogels; (D) Number of junctions of hydrogels.

As a wound application material, biocompatibility such as cell compatibility is an important indicator for evaluating the quality of hydrogels. The cytotoxicity of hydrogels toward L929 fibroblasts was evaluated through CCK‐8 cell viability assay. The results demonstrate the cell survival rate of L929 fibroblasts in all hydrogel extracts were over 95% (Figure 4A). This indicates that all hydrogels in our work were nontoxic and exhibit excellent cell compatibility. The groups with the addition of GA, AS, or both hydrogel extracts showed a survival rate of over 100%, indicating that they have the activity of promoting cell proliferation, among which AS has the strongest pro‐proliferation activity.

Angiogenesis accompanies the wound healing process, primarily through the growth of pre‐existing vascular endothelial cells at the wound margin. Proliferation of endothelial cells and enhanced angiogenesis provide oxygen for wound healing, and thus, angiogenesis plays a key role in the formation of nascent granulation tissue. Our findings revealed that, after 4 h, both the GelMA and GelMA/Lip@GA hydrogel groups exhibited a moderate promotion of endothelial cell tubule formation. However, the GelMA/Lip@AS and GelMA/Lip@GA@AS groups displayed significantly enhanced tube formation. The length and the number of junctions showed significantly increase, compared to the control group (Figure 4B–D). These suggest that GelMA/Lip@GA@AS can effectively release drugs into wounds and stimulate angiogenesis in vitro. Interestingly, when GA and AS were used in combination, both the tube length and the number of junctions were higher than when they were used alone, indicating that there may be a synergistic effect between two drugs.

### 3.5 Antimicrobial Property of the Hydrogel

**FIGURE 5:**
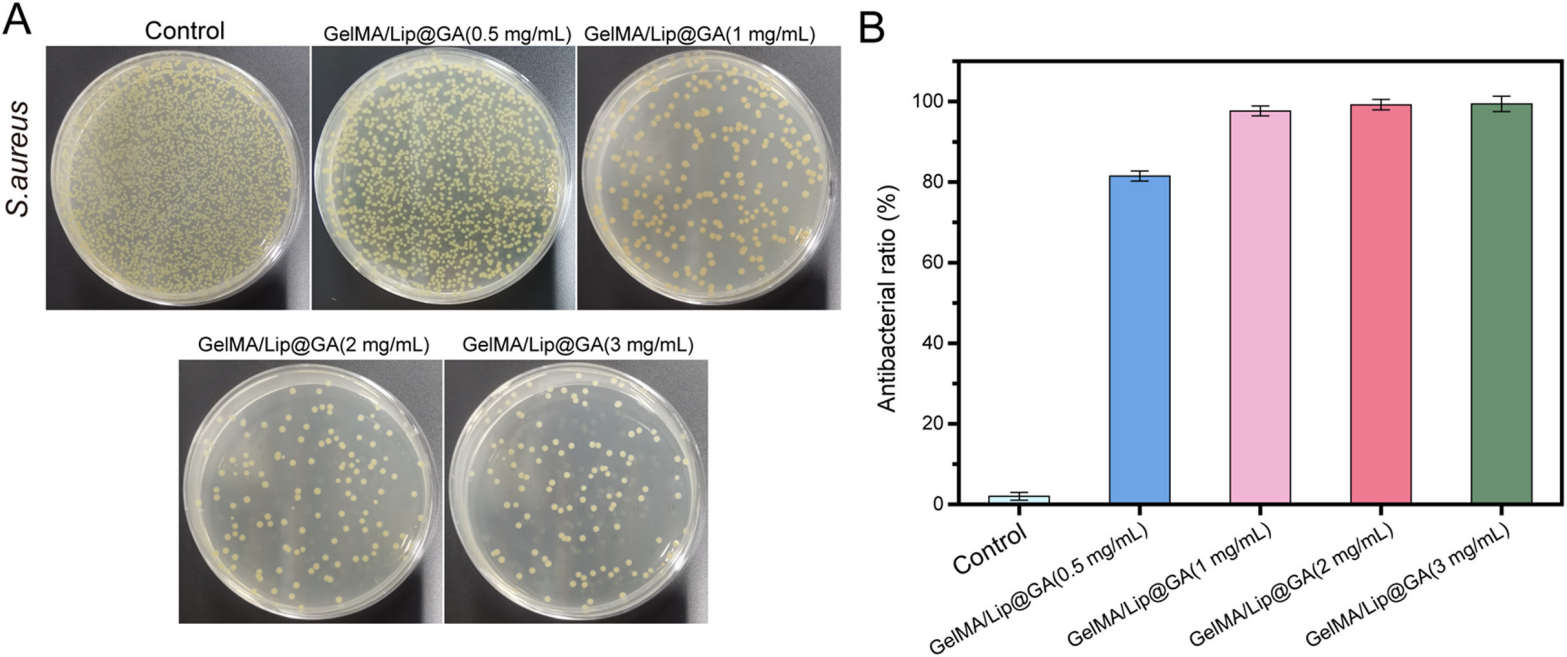
(A) Plate colony growth of four concentrations of GelMA/Lip@GA compared to control; (B) Quantitative comparison of antimicrobial properties of GelMA/Lip@GA compared to control.

The antimicrobial capacity of the hydrogel is essential, as it promotes healing while protecting the wound from pathogenic bacteria infection. *Staphylococcus aureus* (*S. aureus*) is a member of the skin microbiota, commonly coexists as a commensal bacterium and serves as a significant pathogen responsible for wound infections [31]. Here, we respectively tested the growth of *S. aureus* at concentrations of 0.5, 1, 2, and 3 mg/mL of GelMA/Lip@GA, to assess the antimicrobial capacity of the hydrogels. It was found that all four concentrations of hydrogels exhibited significant antimicrobial properties compared to the control group. The number of colonies on the plates decreased (Figure 5A). However, there was no significant difference between the concentrations of 1, 2, and 3 mg/mL (Figure 5B). Collectively, these findings indicate that GelMA/Lip@GA@AS can effectively inhibit the colonization of *S. aureus* and the concentration of 1 mg/mL has sufficient antibacterial effect.

## 4 Conclusion

This study synthesized liposomes loaded with GA and AS by film dispersion‐ultrasonication method, which were then incorporated into a GelMA solution and cross‐linked by ultraviolet light to form a bioactive composite hydrogel for wound dressings. The hydrogel polymer exhibited a porous structure conducive to nutrient transport and gas exchange. Compared with GelMA hydrogel (swelling rate 69.8% ± 5.7%), the swelling rate of GelMA/Lip@GA@AS was 52.1% ± 1.0%, which is relatively better. In addition, GelMA/Lip@GA@AS also has better compression and rheological properties, and the in vitro biodegradability was not significantly different from that of the collagenase‐treated group. In vitro experiments show that the hydrogel polymer has a stable drug release rate, good biocompatibility, and promoting angiogenesis effect. In the face of wound infection, the antibacterial ability of hydrogel polymer was evaluated, and the results show that GelMA/Lip@GA@AS at concentrations of 0.5, 1, 2, and 3 mg/mL could inhibit the growth of *Staphylococcus aureus*. These findings indicate the potential of GelMA/Lip@GA@AS hydrogel as an innovative wound dressing material.

## 5 Discussion

The skin serves as the body's primary line of defense, consistently exposed to the external environment, and its barrier function is crucial for maintaining skin homeostasis. Previous studies have found that barrier dysfunction can trigger psoriasis, seborrheic dermatitis, and various other skin disorders [32]. Psoriasis is a common chronic papulosquamous skin disease that is associated with several significant medical conditions, including depression, psoriatic arthritis, and metabolic syndrome [33]. Previous studies have demonstrated the potential of using a combination of curcumin and GA for the synergistic treatment of psoriasis [34]. Furthermore, in traditional Chinese medicine, *Centella asiatica* is well known for its anti‐psoriasis properties, as it has been shown to inhibit the proliferation of SVK‐14 keratinocytes [35]. Seborrheic dermatitis is also a prevalent chronic skin condition, typically manifesting as an inflammatory response to *Malassezia* species, and it tends to occur in seborrheic areas, such as the scalp, face, and back [36]. Clinically, creams containing GA have been used to treat facial seborrheic dermatitis in HIV‐positive patients [37]. Therefore, this raises the question of whether GelMA/Lip@GA@AS hydrogel may not only enhance wound healing in damaged skin but also provide synergistic therapeutic effects for skin diseases. In addition, due to the existing conditions, the relevant experiments in this article have only been conducted in vitro, without an in vivo model. In the future, this hydrogel may be applied to wound sites through transdermal injection or other methods, but the dose range, precise delivery to the target area, and potential toxic side effects on living organisms need further exploration.

## Conflicts of Interest

The authors declare no conflicts of interest.

## Data Availability

Data will be made available on request.
